# Social Condition and Cognitive Function Among Boston Area Puerto Ricans

**DOI:** 10.1002/alz.087388

**Published:** 2025-01-09

**Authors:** Natalia Palacios, Shishova Ekaterina, Tammy Scott, Luis Falcon, Katherine L Tucker

**Affiliations:** ^1^ Zuckerburg College of Health Sciences, University of Massachusetts Lowell, Lowell, MA USA; ^2^ University of MA, Lowell, Lowell, MA USA; ^3^ Tufts University, Boston, MA USA; ^4^ Univ. of MA, Lowell, Lowell, MA USA; ^5^ University of New Hampshire, Lowell, MA USA

## Abstract

**Background:**

This study aimed to assess associations between socioeconomic condition and cognitive function among Puerto Rican adults residing in the greater Boston Area.

**Methods:**

We assessed the relationship between a score of social condition, encompassing education, income‐to‐poverty ratio, perceived stress, food security, and psychological acculturation, and cognitive function in a cohort of Puerto Rican Adults residing in the greater Boston area. The score was assessed over more than 12 years of follow‐up and ranged from 0 (best social condition) to 15 (poorest social condition). We examined the varying contributions of traditional (education and income) vs. psychosocial (perceived stress, food security, and psychological acculturation) in relation to cognitive function.

**Results:**

After adjusting for covariates, poorer 12‐year average social condition was associated with poorer cognitive function (β = ‐ 0.52, 95% CI = [‐ 0.66, ‐ 0.38; *P* trend = 5.7×10^−14^ for highest vs. lowest disparity category). The association was stronger for traditional (β = ‐ 0.53, 95% CI = [‐ 0.67, ‐ 0.40] *P* trend = 5.7×10^−14^) than psychosocial (β = ‐0.20, 95% CI = [‐ 0.34, ‐ 0.06] *P* trend = 0.05) measures, but both were significantly associated with cognitive function.

**Conclusions:**

Our findings in US‐mainland Puerto Rican adults suggest that socioeconomic condition, both traditional and psychosocial, are associated with cognitive function. This work highlighting the importance of addressing and reducing social disparities to promote better cognitive health outcomes.

